# Supplementary material for: Population Response of Rhizosphere Microbiota of Garden Pea Genotypes to Inoculation with Arbuscular Mycorrhizal Fungi
Source: Int J Mol Sci. 2023 Jan 6;24(2):1119. doi: 10.3390/ijms24021119 (PMC9866347; doi:10.3390/ijms24021119)
Supplement: Supplementary file 1 [file ijms-24-01119-s001.zip › Supplement 3_media_compositions.pdf]

Supplement 3

Table S2. Isolation media compositions

Isolation media	Compositions (g L^{-1})
1. Ammonium mineral salts medium	Soluble starch, 10.0; Ammonium sulphate, 1.0; Dipotassium hydrogen phosphate, 1.0; Magnesium sulphate, 1.0; Calcium carbonate, 3.0; Sodium chloride, 1.0; Agar, 25 ; Final pH 6.8 – 7.2.
2. Beef extract-glucose agar medium	Peptone, 10.0; Beef extract, 10.0; Sodium chloride, 5.0; Dextrose, 5.0; Agar, 20.0; Final pH 7.0±0.2.
3. Gause's synthetic agar medium	Soluble starch, 20.0; Sodium chloride, 0.5; Ferrous sulfate, 0.01; Potassium nitrate, 1.0; Sipotassium hydrogen phosphate, 0.5; Magnesium sulfate, 0.5; Agar, 15, Final pH 7.3±0.2.
4. Czapek Dox Agar	Sucrose, 30.0; Sodium nitrate, 2.0; Dipotassium phosphate, 1.0; Magnesium sulphate, 0.5; Potassium chloride, 0.5; Ferrous sulphate, 0.01; Agar 30.0; Final pH 6.8 – 7.2.
5. Ashby's Mannitol agar	Mannitol, 20.0; Dipotassium hydrogen phosphate, 0.2; Magnesium sulphate, 0.2; Sodium chloride, 0.2; Potassium sulphate, 0.1; Calcium carbonate, 5.0; Agar, 15.0; Final pH 7.4±0.2.
6. Pikovskayas Agar	Yeast extract, 0.5; Dextrose, 10.0; Calcium phosphate, 5.0; Ammonium sulphate, 0.5; Potassium chloride, 0.2; Magnesium sulphate, 0.1; Manganese sulphate, 0.0001; Ferrous sulphate, 0.0001; Agar, 15.0; Final pH 7.0 ± 0.2
7. Yeast Mannitol Agar w/ Congo Red	Yeast extract, 1.0; Mannitol, 10.0; Dipotassium phosphate, 0.5; Magnesium sulphate, 0.2; Sodium chloride, 0.1; Congo red, 0.025; Agar, 20.0; Final pH 6.8±0.2.
