# Supplementary figures and images for: Population Response of Rhizosphere Microbiota of Garden Pea Genotypes to Inoculation with Arbuscular Mycorrhizal Fungi
Source: Int J Mol Sci. 2023 Jan 6;24(2):1119. doi: 10.3390/ijms24021119 (PMC9866347; doi:10.3390/ijms24021119)

## Supplement 2

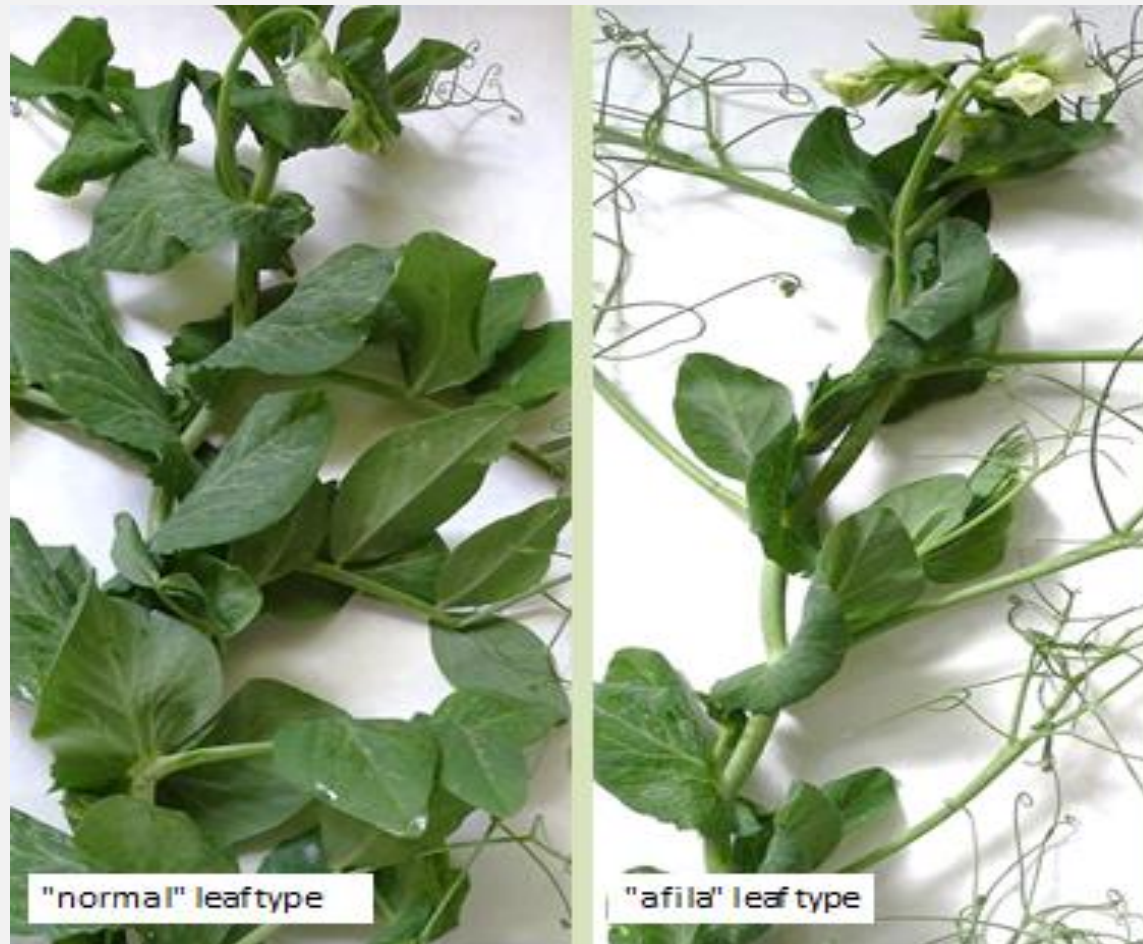

**Figure S1.** Photos of habitus of garden pea genotypes with “normal” and “afila” leaf types

Supplement: Supplementary file 1 [file ijms-24-01119-s001.zip › Suplement 2-photos of pea genotypes.pdf]
